# Human Alveolar Echinococcosis in Kyrgyzstan

**DOI:** 10.3201/eid1907.121405

**Published:** 2013-07

**Authors:** Jumagul Usubalieva, Gulnara Minbaeva, Iskender Ziadinov, Peter Deplazes, Paul R. Torgerson

**Affiliations:** Government Sanito-Epidemiology Unit, Bishkek, Kyrgyzstan (J. Usubalieva, G. Minbaeva);; University of Zurich, Zurich, Switzerland (I. Ziadinov, P. Deplazes, P.R. Torgerson)

**Keywords:** Echinococcus multilocularis, emergence, Alveolar echinococcosis, Kyrgyzstan, Central Asia, tapeworms, parasites, zoonoses

## Abstract

Human echinococcosis is a reportable disease in Kyrgyzstan. Between 1995 and 2011, human alveolar echinococcosis increased from <3 cases per year to >60 cases per year. The origins of this epidemic, which started in 2004, may be linked to the socioeconomic changes that followed the dissolution of the former Soviet Union.

Alveolar echinococcosis (AE) is a devastating disease in humans, caused by the larval stage of the fox tapeworm, *Echinococcus multilocularis* (*1*). In the absence of treatment, the condition is often fatal, although expensive and successful treatment options are available and have recently been documented to be effective (*2*). The closely related parasite *E. granulosus,* commonly transmitted between dogs and livestock, causes cystic echinococcosis (CE) when it infects humans. CE has emerged throughout central Asia following the dissolution of the former Soviet Union and has been attributed to changes in animal husbandry practices, decline in veterinary public health services, and increases in dog populations (*3*). We report evidence of an emerging epidemic of human AE in the former Soviet Republic of Kyrgyzstan.

## The Study

Echinococcosis is notifiable in Kyrgyzstan, a small, mountainous, central Asian country (199,900 km^2^) of ≈5.5 million inhabitants. All confirmed diagnoses are reported to the Government Sanito-Epidemiology Unit in Bishkek. Cases are reported as either CE or AE by morphologic and histologic examination of resected lesions after surgical treatment. Age, sex, origin, and occupation of patients with reported cases are recorded. This reporting procedure has been used since the time of Soviet administration. However, the number of cases is likely underreported, and many case-patients with echinococcosis do not receive treatment because of widespread poverty and misdiagnoses. We analyzed the official reported cases of AE for the years 1995 through 2011 and present the annual numbers of all case-patients, categorized by sex and district of origin.

**Figure 1:**
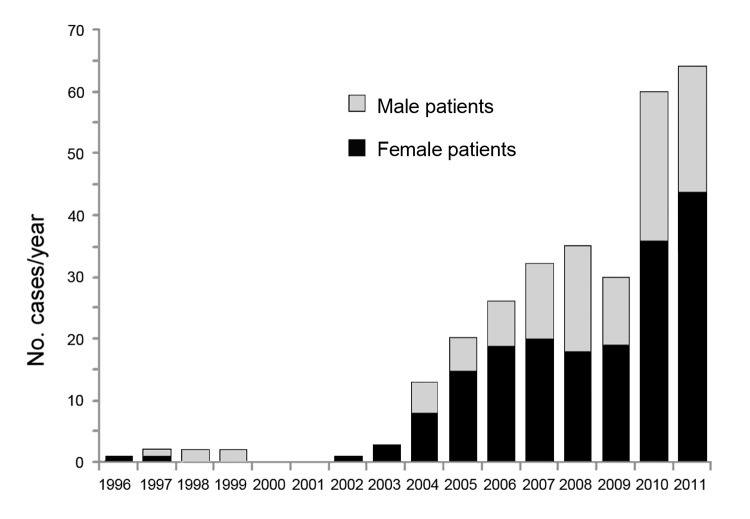
Number of alveoloar echinococcosis cases reported in Kyrgyzstan, by patient sex, 1995–2011.

**Figure 2:**
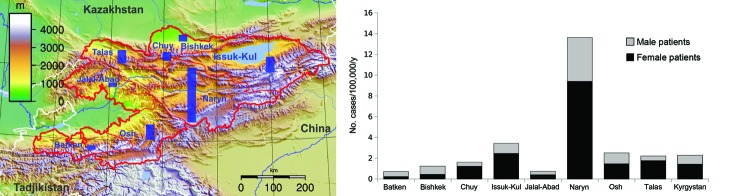
Relative incidence of alveoloar echinococcosis in Kyrgyzstan, by district, 1995–2011. The size of the bars on the map is proportional to the incidence.

After the first AE case was recorded in 1996 in Kyrgyzstan, 0–3 cases occurred each year until 2003. Since 2004, the numbers of reported cases have increased substantially, reaching 61 in 2011 (Figure 1) (p<0.0001, χ^2^ for trend). The total number of AE cases reported from 1995 through 2011 was 291. Of these, 185 case-patients were female and 106 case-patients were male (p<0.0001, χ^2^). The mean age of case-patients was 33.4 years, with a range of 3–76 years. Eight cases occurred in children <10 years of age, and an additional 30 cases occurred in children 10–19 years of age (Table). The highest incidences were from the Osh, Issyk-Kul, and Naryn Oblasts (a subnational administrative division); the annual incidence in Naryn for the last 2 years of the study period was 7.1 cases per 100,000 population (Figure 2). All other districts had a mean annual incidence of <2 cases per 100,000, whereas the mean annual incidence nationwide is 1.2 cases per 100,000 with relatively few cases from the Batken and Talas Oblasts (Figure 2).

**Table:** Distribution of alveolar echinococcosis cases by patient age and sex, Kyrgyzstan, 1995–2011

Age, y	No. patients		Total
	Male	Female	
<10	5	3	8
10–19	13	17	30
20–29	38	59	97
30–39	23	50	73
40–49	16	25	41
50–59	8	21	29
≥60	3	10	13

These data suggest an epidemic, at least as far as confirmed reported cases indicate, of AE in Kyrgyzstan that began in ≈2004. The case-patients reported here were also young (mean age 33 years) compared to case-patients in Europe, for example, in Switzerland, where the higher mean age is 54 years (*2*). Kyrgyzstan, however, has a young population; median age is 24.7 years, whereas the median age of those infected in Switzerland is 41.3 years (data from US Census Bureau, International Programs, www.census.gov/population/international/data/idb/informationGateway.php). This difference in the population pyramids means that Kyrgyzstan has a much higher proportion of children and young adults than Switzerland, and therefore a greater proportion of Kyrgyz case-patients would have been exposed and diagnosed at an earlier age.

## Conclusions

Dogs are usually essential for CE transmission, and thus they are the key to the increasing human CE incidence reported previously (*3*). Dogs are highly susceptible to infection with *E. multilocularis* (*4*), and 18% of dogs from Naryn Oblast harbor this parasite, a similar prevalence to *E. granulosus* infection (*5*). Therefore, contact between humans and dogs responsible for transmission of CE could result in transmission of AE.

More than 90% of the estimated 18,000 annual new cases of AE throughout the world are in China, mainly the Tibetan plateau (*6*), where there is also a concomitant high prevalence of *E. multilocularis* infection in dogs (*7*). Dog contact has been reported as a risk factor for human AE on the Tibetan plateau (*8*). Therefore, *E. multilocularis* infection in dogs in Kyrgyzstan may be key in the development of the reported epidemic of human AE. The parasite has colonized dogs because dog populations have increased and because dogs scavenge for rodents. Dogs that spend a large proportion of time untied, and thus are able to roam, have a higher prevalence of *E. multilocularis* infection in Kyrgyzstan (*5*). 

This epidemic also appeared 10–15 years after the dissolution of the Soviet Union. This time frame coincides with the hypothesized latent period of human AE. In Switzerland, human AE has also emerged, but this has been linked to an increase in the fox population (*9*). Unlike the situation in Switzerland, we have no data on fox populations and thus cannot speculate about any possible changes in fox and small animal ecology that could be an alternative hypothesis for changes in AE incidence. However, the recent incidence in Kyrgyzstan is 4–5 times higher than it is in Switzerland, and therefore the epidemiology of transmission to humans is likely to be different. However, among foxes in Kyrgyzstan, prevalence of infection with *E. multilocularis* is 65%, with a mean abundance of >8,500 parasites per fox (*10*), and this abundance of parasites may be critical in transmission to humans if substantial fox–human contact occurs. Nevertheless, *E. multilocularis* infection in foxes in Kyrgyzstan is unlikely to be a recent phenomenon because *E. multilocularis* was reported in rodent intermediate hosts in the 1950s (*11*).

Lithuania, another former Soviet republic, also reported increased numbers of AE cases during 1997–2006 (*12*). In Lithuania, involvement of dogs in the transmission of *E. multilocularis* has been suggested. There is also a report of human AE emergence in Poland (*13*), another former communist country, with a steady increase in cases from 1990 to 2011. In Poland, the fox population is increasing, and many AE cases originate in areas with large fox populations. The role of dogs in transmission in Poland cannot yet be clarified because of lack of data.

This report does not prove that human AE has increased in Kyrgystan. It is possible that reporting of cases may have improved. Nonetheless, AE has long been known in the states of the former Soviet Union; cases were diagnosed in Russia as early as 1900 (*14*). Thus it seems unlikely that a failure to diagnose large numbers of AE cases would have occurred, which would have to have been the situation if the incidence of human AE had remained constant. Furthermore, medical services were adversely affected during a period of severe economic hardship after the dissolution of the Soviet Union, thus making improved diagnosis an unlikely factor in the increased number of cases reported. In the disease-endemic area in central Europe, the numbers of reported cases of AE have increased since the disease was first described in the mid 19th century, and this increase may be due to improved diagnosis in recent decades (*14*). Increases in fox populations, particularly urban fox populations, may have provided the environment for increased transmission to humans (*15*), but to date, the only convincing evidence that this increase in fox populations has resulted in increased numbers of AE cases has come from Switzerland (*9*). In conclusion, AE appears to be a rapidly emerging problem in Kyrgyzstan, which may be related to profound socioeconomic changes that have occurred in the past 25 years.
