# Correction: Vol. 19, No. 7

**DOI:** 10.3201/eid2003.C22003

**Published:** 2014-03

**Keywords:** errata, erratum, correction

The article Human Alveolar Echinococcosis in Kyrgyzstan (J. Usubalieva et al.) incorrectly labeled the *y*-axis of Figure 1. The corrected figure and caption are reproduced here, and the article has been corrected online (wwwnc.cdc.gov/EID/article/19/7/12-1405_article.htm).

**Figure 1:**
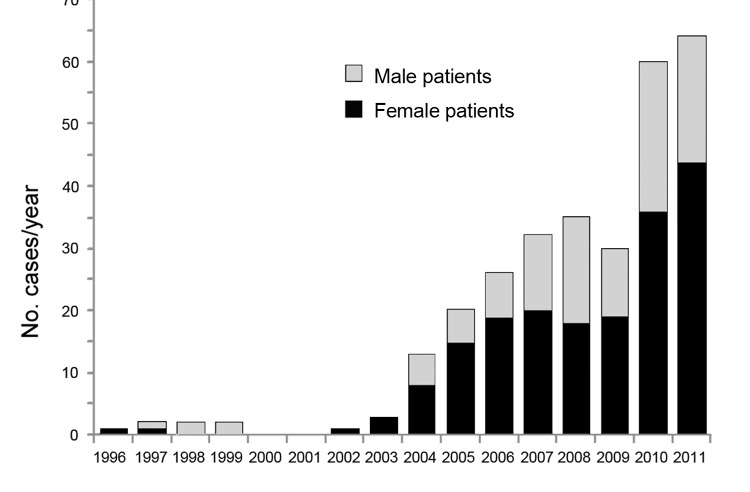
Number of alveoloar echinococcosis cases reported in Kyrgyzstan, by patient sex, 1995–2011.

